# Pathophysiological Evaluation of Cerecyte Coil Embolization for Experimental Broad Neck Aneurysms

**DOI:** 10.5402/2012/137873

**Published:** 2012-06-26

**Authors:** Kazuhisa Iwamoto, Akira Kurata, Sachio Suzuki, Taketomo Ohmomo, Shigenobu Nakayama, Shigeyoshi Maruyama, Mamoru Takagi, Shingo Konno, Kuniaki Nakahara, Kiyotaka Fujii, Yoshie Yasui, Keiichi Iwabuchi

**Affiliations:** ^1^Department of Neurosurgery, Kitasato University School of Medicine, 1-15-1 Kitasato, Minami-ku, Kangawa, Sagamihara 228, Japan; ^2^Center for Genetic Studies of Integrated Biological Functions, Kitasato University School of Medicine, 1-15-1 Kitasato, Minami-ku, Kangawa, Sagamihara 228, Japan; ^3^Department of Pathology, Kitasato University School of Medicine, 1-15-1 Kitasato, Minami-ku, Kangawa, Sagamihara 228, Japan

## Abstract

Cerecyte second-generation coils feature inner surfaces coated with an absorbable polyglycolic acid (PGA) polymer. Their use is expected to accelerate aneurysm organization, but time course data are limited. The present experimental study was therefore conducted to clarify the processes by pathological examination. *Methods*. Two types of experimental aneurysms were initially generated in adult mongrel dogs, one bifurcation and another of lateral wall type. Long-term persistence of each was defined by follow-up angiography for more than 1 year. Embolization of the aneurysms was then performed using only cerecyte coils, and follow-up angiography was conducted after 2 and 4 weeks followed by pathological examination. *Results*. Organization of both types of broad neck aneurysm was apparent 4 weeks after embolization, which is earlier as compared with already reported data for bare coils.

## 1. Introduction

Coil embolization has become a standard therapeutic approach for cerebral aneurysms, along with clipping surgery. However, long term efficacy of bare coils is not satisfactory. Especially, coil compaction for broad neck aneurysms more than 4 mm in diameter may occur at high frequency, resulting in a requirement for addition embolization [1, 2].

Second-generation coils have been developed which now facilitate early organization of aneurysms. For example, matrix coils coated with polyglycolic-polylactic acid (PGLA) on their outer surfaces are associated with organization as early as 2 weeks after embolization with experimental aneurysms [3].

Cerecyte coils are another type of second-generation coil, featuring an inner surface coat of absorbable polyglycolic acid (PGA) polymers [4]. Since pathological examination of the time course of changes with its use for aneurysmal embolization has not been reported, the present experimental study was conducted.

**Methods**

Adult 5 female mongrel dogs, with body weights ranging from 12.5 to 20.5 kg, were initially intubated and underwent operations under general anesthesia with halosen. Two types of experimental broad-neck aneurysms (neck size: more than 4 mm) were generated, one of bifurcation aneurysm and another of lateral wall type. Bifurcation type aneurysms were made as follows. Initially, a contralateral common carotid artery was anastomosed to the ipsilateral carotid artery and thereafter a venous pouch developed, as already reported in detail [5]. Long-term persistence of each aneurysm was defined by follow-up angiography more than 1 year after the aneurysmal creation. Embolization was then performed using only cerecyte coils and follow-up angiography was conducted after 2 and 4 weeks, followed by pathological examination. Four bifurcation aneurysms and two lateral wall types of aneurysms were made. In one dog, both types of aneurysms were made; all of the aneurysms were followed by angiography more than 2 years after the creation and defined as persistence of the aneurysm.

## 2. Endovascular Surgery

**Figure 1:**
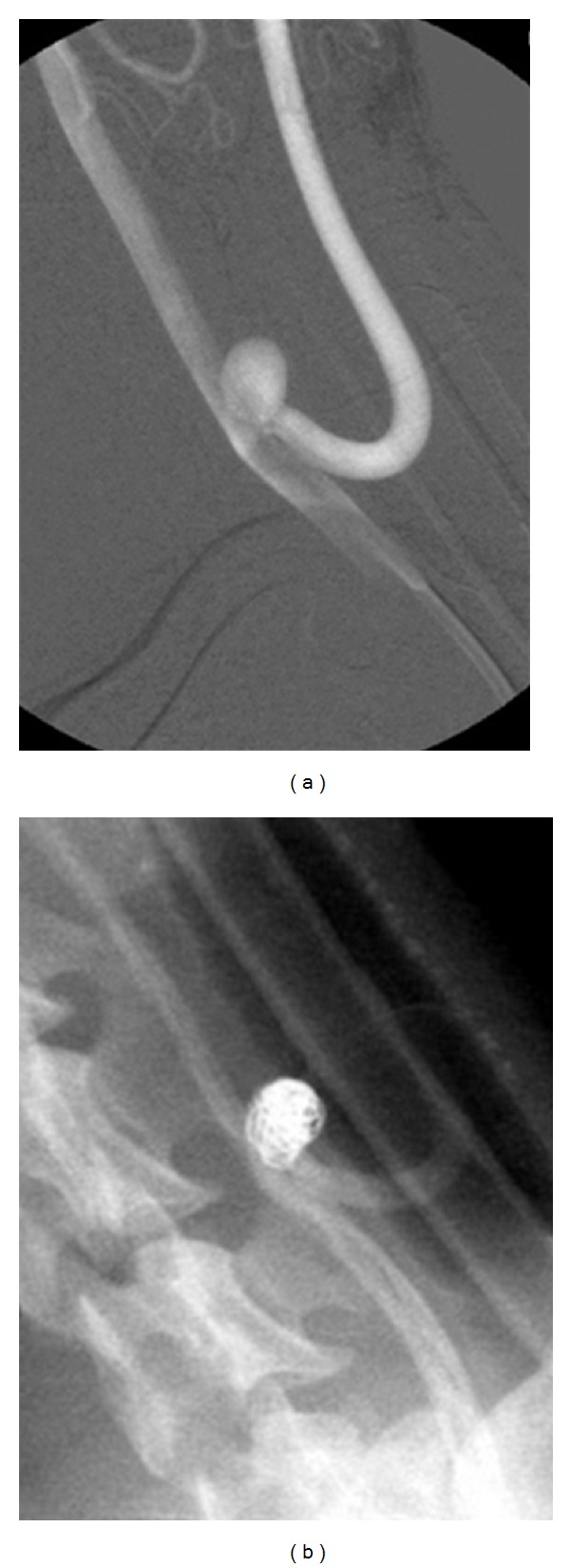
Angiograms of a bifurcation type aneurysm. (a) Pre-embolization. (b) Three weeks after embolization, showing occlusion of the aneurysm.

**Figure 2:**
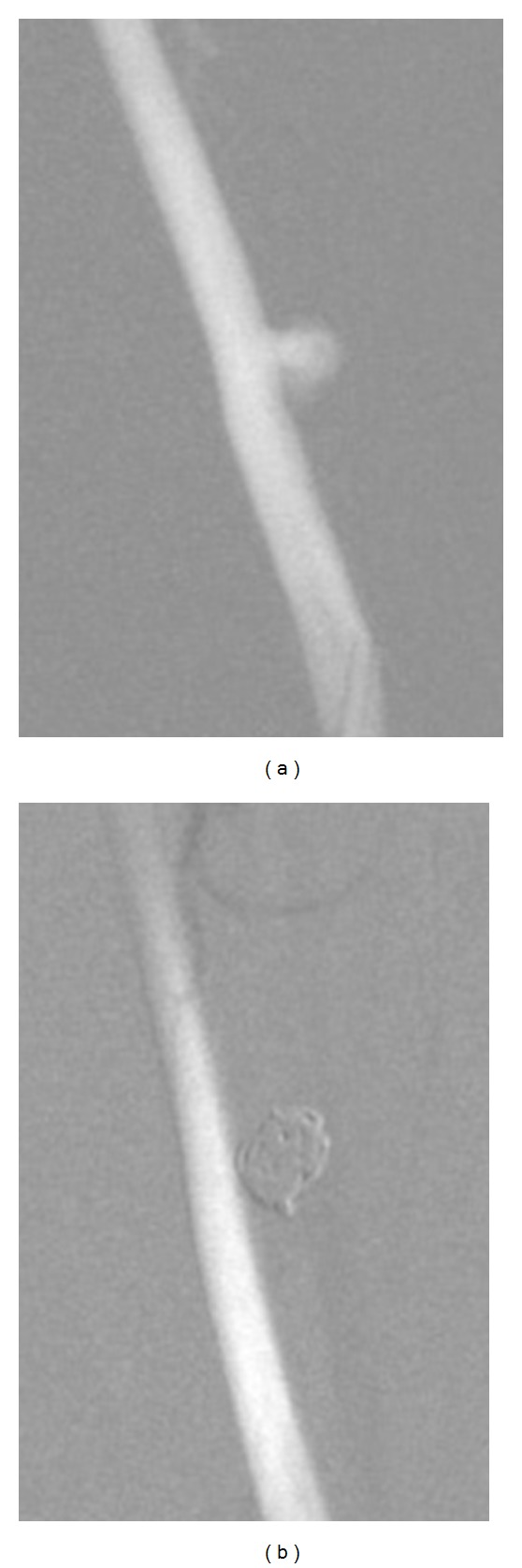
Angiogram of a lateral wall type aneurysm. (a) Pre-embolization. (b) Two weeks after the embolization, showing complete occlusion of the aneurysm.

Initially, a 6F sheath was inserted into the right femoral artery and heparinization was started with a bolus injection of 2000 U heparin. Then a microcatheter (SL 10, Boston Scientific USA) preceded by a microguide wire (Agility 10 type, J & J, USA) was introduced into the aneurysm under roadmapping and thereafter complete embolization was achieved using cerecyte coils (Presidio, Ultipaq, Micrus, USA). Follow-up angiography was conducted after one to 4 weeks for bifurcation and 2 and 4 weeks for lateral wall aneurysms (Figures 1 and 2). For one lateral wall aneurysm further coil embolization was added because of coil compaction 1 week after the embolization.

## 3. Pathophysiological Examination

Aneurysms were removed immediately after the follow-up angiography and fixed with 10% formalin. The specimens were routinely processed for embedding in Metakril acid MMA and thereafter sectioned by microcutting machine as 30–40 *μ*m. Pathologist evaluated the pathological findings.

## 4. Results 

**Figure 3:**
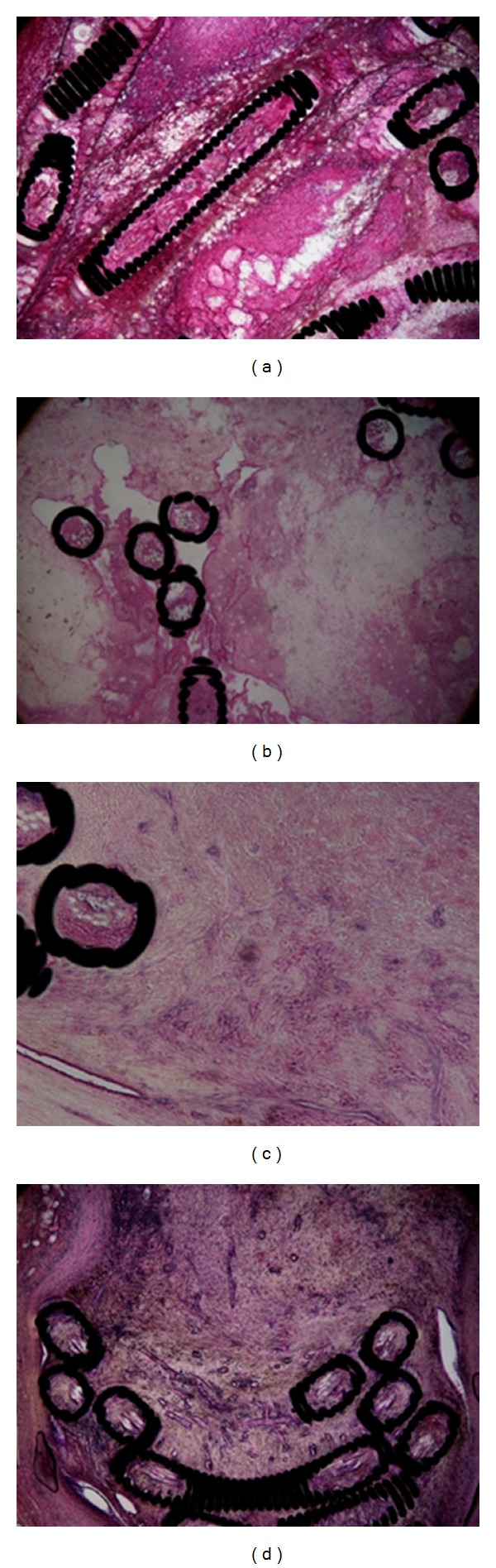
Photomicrographs showing a bifurcation type aneurysm. Hematoxylin and eosin staining. (a) Pathological findings 1 week after the embolization showing no organization. (b) Pathological findings 2 weeks after the embolization showing no organization. (c) Pathological findings 3 weeks after the embolization showing partial organization. (d) Pathological findings 4 weeks after the embolization showing organization of the whole aneurysm.

**Figure 4:**
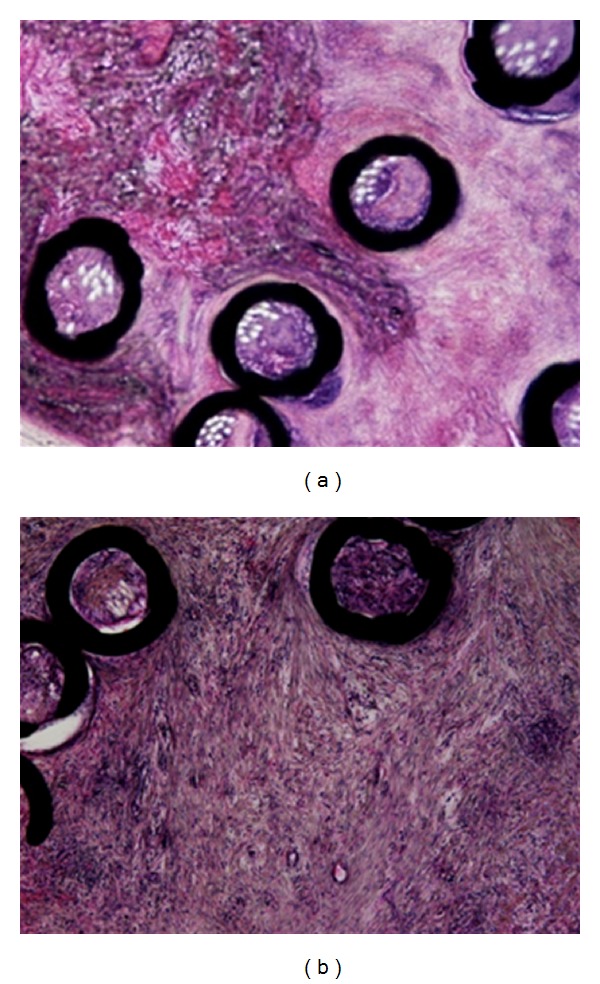
Photomicrographs of a lateral wall type aneurysm. Hematoxylin and eosin staining. (a) Pathological findings 2 weeks after the embolization showing partial organization. (b) Pathological findings 4 weeks after the embolization showing organization of the whole aneurysm.

Bifurcation aneurysms demonstrated fresh thrombi 1-2 weeks after the embolization, but organization was not yet evident. From 3 weeks, however, partially organization started, becoming total after 4 weeks (Figure 3). Lateral wall aneurysms demonstrated organization 2 weeks after the embolization and this became total after 4 weeks (Figure 4).

## 5. Discussion

Aneurysmal coil embolization is already well established as an effective treatment, but coil compaction frequently occurs, especially with broad neck and large aneurysms [1] resulting in reopening [6]. Second-generation matrix and cerecyte coils have been developed to overcome this problem, and an earlier experimental study using bare coil of GDC reported aneurysm organization within two months of embolization [2]. In fact, matrix coils coated with absorbable PGLA polymer on the outer surface allowed organization after 2 weeks [3]. Therefore, cerecyte coils coated with polyglycolic acid on the inner surfaces were developed in the expectation that they would also provide early organization of aneurysms compared with bare coils [4]. Hydrocoils coated with hydrogel have a high embolization ratio resulting in a low recanalization rate [7] and Yoshino et al. reported that aneurysms embolized with cerecyte coils in an experimental study showed organization after three months [8]. However, an actual pathological study with regard to cerecyte coils has hitherto not been reported. In the present investigation, the lateral wall type of aneurysm embolized with cerecyte coils showed early organization 2 weeks after embolization, while organization of intra-aneurysmal thrombi was evident after three weeks with bifurcation aneurysms. The latter time delay was presumably due to direct inflow in the bifurcation aneurysm.

Pig and dog models are available for experimental aneurysms [1–3, 5]. Mason and Read [9] reported the pig to have high coagulability and low fibrinolysis while the dog has the highest fibrinolytic tendency. Raymond et al. reported [10] experimental aneurismal study of dogs showed occurrence of thin internal membrane, but pig study easily induced thick internal membrane resulting in no tendency of recanalization.

In this study, to avoid natural occlusion and the tendency for easy organization, we used canine broad neck aneurysms with long term patency more than 2 years after their creation.

Both lateral wall and bifurcation type aneurysms were totally occluded with total organization 4 weeks after the embolization, much earlier compared with using bare coils. Since antiplatelet drugs to prevent thrombotic events are clinically applied, which may influence time delay of aneurysmal organization, further experimental studies are now planned in our laboratory to assess this question, comparing bare with second-generation coils in the same dogs. 

## 6. Conclusion

In this experimental study, cerecyte second-generation coils were suggested to provide earlier organization of aneurysms as compared with reported results with bare coils.
